# Determinants of timely uptake of ITN and SP (IPT) and pregnancy time protected against malaria in Bukoba, Tanzania

**DOI:** 10.1186/s13104-016-2122-3

**Published:** 2016-06-21

**Authors:** Joyce Protas, D. Tarimo, C. Moshiro

**Affiliations:** Department of Community Health Nursing, Hubert Kairuki Memorial University, P.O. Box 65300, Dar Es Salaam, Tanzania; Department of Parasitology, Muhimbili University of Health and Allied Science, Dar Es Salaam, Tanzania; Department of Epidemiology and Biostatistics, Muhimbili University of Health and Allied Science, Dar Es Salaam, Tanzania

**Keywords:** Malaria in pregnancy, Prevention, Determinants of timely uptake, Insectcides treated nets, Sulfadoxine-pyrimethamine

## Abstract

**Objective:**

Insecticides treated nets (ITNs) and intermittent preventive therapy with two doses of sulfadoxine-pyrimethamine (SP IPTp) are the cornerstone for malaria control in pregnancy. Despite the coverage of these interventions being high, it is not known whether they confer optimal protection time against malaria in pregnancy. This study investigated the timing and determinants of timely uptake of SP(IPTp) and ITNs and the pregnancy time protected.

**Methods:**

A health facility based cross-sectional study was carried out in Bukoba urban district from 16th April to 29 May 2013. Involving pregnant women and post natal mothers attending Reproductive and Child Health (RCH) clinics. Data on their socio-economic background, pregnancy history and attendance to RCH, receipt of a voucher and acquisition of an ITN as well as SP for IPTp were collected. Their responses were validated from the records of antenatal cards. Data was analysed using SPSS computer program version 20.

**Results:**

A total of 530 mothers were recruited. The overall uptake of SP IPTp was 96 % and the uptake of two SP (IPTp) doses was 86 %. Timely uptake of 1st dose was predicted by early antenatal booking, [AOR 2.59; 95 % CI 1.51–4.46; P = 0.001], and the availability of SP at the facility [AOR 4.63; 95 % CI 2.51–8.54; P < 0.0001]. Uptake of 2nd dose was independent of any predictor factors. A total of 486 (91.6 %) women received ITNs discount vouchers at different gestational age and of these, less than a quarter (21.4 %) received timely. Timely receipt of discount voucher was highly predicted by early antenatal booking [AOR 200; 95 % CI 80.38–498; P < 0.0001].

**Conclusion:**

Although there is a high coverage of SP IPTp and discount vouchers for ITNs, timely uptake and therefore optimal protection time depended on early antenatal booking, the availability of (SP IPTp) and discount voucher at the health facility.

## Background

The World Health Organization (WHO) currently recommends and emphasizes the use of: insecticide-treated nets (ITNs), sulfadoxine-pyrimethamine intermittent preventive treatment (IPT), effective case management of malaria and anemia; as a package of interventions for controlling malaria during pregnancy in areas with stable (high) transmission of *Plasmodium falciparum* [1]. In areas of stable *P. falciparum* transmission, prevention of asymptomatic malaria infection through a two-pronged approach of SP(IPTp) and ITNs results in the greatest health benefits; such us reduction in infant born with low birth weight [1].

The facts that in most African countries, over 80 % of pregnant women make multiple antenatal clinic visits provides a major opportunity for prevention of malaria, along with other priority diseases affecting pregnant women [1, 2].

The National Malaria Control Programme (NMCP) for Tanzania adopted the WHO recommendations for prevention of malaria in pregnancy in 2001. In Tanzania it is a National policy to offer SP(IPTp) to all pregnant women attending antenatal clinics between 16 and 24 weeks for the first SP(IPTp) and between 28 and 32 weeks for the second SP (IPTp) [3]. Also since October 2004, the United Republic of Tanzania’s national ITN strategy [known as the Tanzania National Voucher Scheme (TNVS)] has provided subsidized ITNs targeted to pregnant women and infants [4]. A voucher known as “Hati Punguzo” (Swahili for ‘discount voucher’) with a value of US$3 (75 % of the cost of an ITN) has been distributed to all pregnant women attending antenatal clinic. Partly as a result of this national scheme, nets are widely available throughout Tanzania [5, 6]. Although the coverage of these interventions is high, it is not known whether they confer optimal protection time against malaria in pregnancy. For ITNs to confer optimal protection time an ITN must be obtained in the 1st antenatal clinic visit between 12th and 18th weeks of pregnancy while two SP doses must be received in the 2nd visit between 16th and 24th weeks and in the 3rd visit between 28th and 34th weeks of pregnancy, Therefore, this study investigated the timing and determinants of timely uptake of SP (IPTp) and ITNs during pregnancy, and pregnancy protected time for malaria.

## Methods

### Study area

This study was done in Bukoba Urban District in Tanzania due to it being along the lake Victoria zone with high prevalence of malaria [5].

### Study design

This was a facility based cross-sectional study conducted from 16th April to 29th May, 2013.

### Study population

The study population included pregnant women with ≥36 weeks of gestation and postnatal mothers within 7 days after delivery attending to Reproductive & Child Health (RCH) clinics of three public health facilities. Using the formula z^2^p (100−p)/e^2^, marginal error (e) of 4 %, z at 95 % Confidence interval of 1.96, and prevalence of pregnant women who use the first dose of SP timely of 67(7). The sample size calculated was 530 participants.

### Sampling procedures

#### Selection of the facility

Bukoba urban district has seven government health facilities. Among these facilities, there is only one hospital; Bukoba regional hospital, which was automatically included in the study. Two strata were made on the remaining levels of health facilities, from each strata one facility was randomly selected. These included Kashai dispensary and Zamzam health center.

#### Selection of women in each facility

The allocation of participants per health facility was based on the available information on the average number of pregnant women with ≥36 weeks of gestation and postnatal women attending ANC to these selected facilities. This was four from dispensary, six from health centre and 15 from hospital level. This made the ratio of 4:6:15. Thus among 530 women 85, 127 and 318 were recruited from the dispensary, health canter and hospital respectively. To obtain the required sample size from each health facility, convenience method of sampling was employed.

### Data collection procedure

Trained Research assistants were on selected health facilities from Monday to Friday. Participants were asked a series of closed ended questions about: their socio-economic background, pregnancy history and attendance to antenatal clinic. They were also interviewed on the receipt of a voucher and acquisition of an ITN; and receipt of SP for IPTp as well as reasons for non-use of these interventions. Furthermore, the reported use of SP (IPTp) and uptake of ITN voucher was confirmed by checking the information from antenatal card against their verbal responses.

### Data analysis

Data entry and analysis was carried out using SPSS computer software version 20 and frequency tables were obtained to all study variables. Descriptive analysis was done by using frequencies, percentages and means where appropriate. Association between explanatory variable and the outcome of interest was done using 2 × 2 tables. Bivariate and multivariate logistic regression analyses were used to examine independent variables that influence the outcome variable. Odds Ratios with corresponding 95 % Confidence interval are presented. All independent variable found significant in the bivariate analysis were included in the multivariate analysis. AP value of less than or equal to 0.05 was considered statistically significant.

### Ethical consideration

Ethical clearance was obtained from Muhimbili University of Health and Allied Sciences (MUHAS) Research and Publications committee. Permission to conduct this study in Bukoba urban district was obtained from the Regional Administrative secretary (RAS), District Medical Officer and In charges of the respective facilities. Written consent from the study participant was obtained and confidentiality on their information was highly maintained.

Definition of outcome variable.

- Timely uptake of ITN voucher is a self report; receiving an ITN voucher at 12–18 weeks of gestation.
- Timely uptake of ITN is a self report; redeeming an ITN voucher at 12–18 weeks of gestation.
- Timely uptake of (SP IPTp) is a self report receiving.

First dose of SP at 16th–24th weeks of pregnancy.

Second dose of SP at 28th–34th weeks of pregnancy.

Optimal protection time is the appropriate amount of time for which protection against malaria is conferred by uptake of either of these products.

For SP is between 16 and 24 weeks. (one must receive two doses on time).

For ITN is between 22 and 28 weeks. (one must receive an ITN voucher and redeem for between 12 and 18 weeks of gestation and use it.

## Results

A total of 530 women were recruited into this study: 85 were from Kashai dispensary, 127 from Zamzam health center and 308 from Bukoba regional hospital, 510 were pregnant women and 20 were postnatal mothers within 7 days after delivery. All pregnant women were on their ≥36 weeks of gestation with their ages ranging from 14 to 44 years, and their mean age being 28.8 years (SD ± 5.8). About three quarter had primary school education 351 (66.2 %) and majority were married or cohabitating 431 (81.3 %) Table 1.

**Table 1:** Characteristics of the study population (n = 530)

Characteristics	Number	Percentage
*Age (years)*		
14–17	9	1.7
18–35	469	88.5
36–44	52	9.8
*Occupation*		
Farmer	184	34.7
Petty business	252	47.5
Employed/business	94	17.7
*Level of education*		
No formal education	49	9.2
Primary school education	351	66.2
Secondary school and above	130	24.5
*Marital status*		
Married/cohabiting	431	81.3
Single/widow/separated	99	18.7
*Gravidity*		
Primigravidae	161	30.4
Secondigravidae	137	25.8
Multigravidae	232	43.8
*Parity*		
0	182	34.3
1–3	324	61.1
4+	24	4.5

Around three quarters 392 (74 %) of all women made their first visit after 18 weeks of gestation, the majority, 412 (77.7 %), decided on their own to visit ANC, Two-thirds 353 (66.6 %), walk/travel for less than 30 min to reach their health care facility, and 476 (89.8 %) made three or more antenatal visits Table 2.

**Table 2:** Attendance at antenatal clinics (n = 530)

Characteristics	Number	Percentage
*First visit (time in weeks)*		
≤18 weeks of gestation	138	26.0
>18 weeks of gestation	392	74.0
*Influence to make a visit*		
Self	412	77.7
Husband	96	18.1
Others^a^	22	4.2
*Time of walking to the health facility (minutes)*		
≤30	353	66.6
31–60	160	30.2
>60	17	3.2
*ANC visit*		
One	1	0.2
Two	53	10.0
Three or more	476	89.8

^a^Others include mothers, friends and radio

### Proportion of women who receive (SP IPTp) for intermittent preventive treatment

The majority of respondents 508 (96.0 %) used (SP IPTp) for intermittent preventive treatment during pregnancy, only 22 (4 %) did not use with the reasons being: SP was Out of stock for 18 (78 %) and for the remaining 4 (22 %) due to delay in starting antenatal care visit. Among those who used (SP IPTp) for intermittent preventive; 438 (86 %) used two doses and 70 (14 %) used a single dose. Reasons given for not using two doses included lack of (SP IPTp) at the facility for 60 (86 %) and delay in starting antenatal visit for10 (14 %) women.

### Timing of uptake of SP(IPTp) among women

**Fig. 1:**
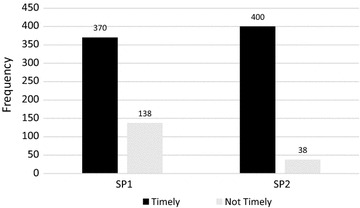
Timing of uptake of SP (IPTp). *SP1* timely uptake first dose of sp; at 16–24 weeks of gestation. *SP2* timely uptake second dose of sp; at 28–34 weeks of gestation

Out of 508 women who received first dose, 370 (72.8 %) received timely, and the mean time for receiving was 21.3 weeks of gestation. Among 438 pregnant women who received second dose; 400 (91.3 %) received timely and the mean time for receiving was 30 weeks of gestation Fig. 1.

### Proportion of women who received discount voucher

Among 530 women, 486 (91.7 %) received discount voucher and 44 (8.3 %) did not; mainly due to its unavailability at the clinic. Of all 486 women who received discount voucher, 104 (21.4 %) received timely and the mean time for receiving discount voucher was at 20.3 weeks of gestation.

### Proportion of women who managed to access ITNs after getting ITN vouchers

Among 486 women who received discount vouchers; 468 (96 %) redeemed for an ITN and 18 (4 %) did not. The reasons given for one not to redeem were; lack of top up money in 6 (33 %) women; 11 (61 %) did not know shops to get an ITN and 1 (6 %) had her own bed net and therefore did not like to use a net obtained from discount voucher scheme because it has a bad smell. Out of 468 who redeemed for an ITN; 63 (13.5 %) redeemed timely and 405 (76.4 %) did not.

### Proportion of women who received optimal protection time after using SP IPTp for intermittent preventive treatment

Of all 530 women, 438 (86 %) received two doses of SP IPTp, from which 340 (77.6 %) received an optimal protection time and 98 (22.37 %) did not.

### Proportion of women who received optimal protection time after using ITN obtained through discount voucher scheme

About 468 women redeemed for an ITN, 63 (13.5 %) received an optimal protection time and 405 (76.4 %) did not.

### Factors associated with timely Uptake of SP (IPTp)

Timely uptake for first dose of SP was determined by: early antenatal booking [OR = 2.59; 95 % CI 1.53–4.438; P = 0.0001], availability of SP at the facility [OR = 4.78; 95 % CI 2.66–8.57; P = 0.0001], multgravidae [1.26; 95 % CI 1.20–1.59; P = 0.05], self decision to attend ANC [OR = 1.59; 95 % CI 1.51–4.47; P = 0.003] Table 3.

**Table 3:** Bivariate analysis on factors associated with timely uptake of first dose of SP (n = 508)

Variable	Total received first dose of n = 508	Timely uptake first dose n = 370	OR (95 % CI)	P value
*Age (years)*				
14–17	7	6 (85.7)	1	
18–35	449	334 (74.4)	0.47 (0.26-1.08)	0.18
36–44	52	30 (57.7)	0.23 (0.026-2.2025)	0.12
*Marital status*				
Not married	95	67 (70.5)	1	
Married	413	303 (73.4)	1.15 (0.70-1.80)	0.53
*Education*				
No formal	45	30 (66.7)	1	
Primary	341	254 (74.5)	1.19 (0.57-2.48)	0.63
Secondary/college	122	68 (70.5)	1.18 (0.52-1.29)	0.39
*Occupation*				
Farmer	177	133 (75.1)	1	
Petty business women	244	180 (73.8)	0.67 (0.39-1.14)	0.14
Business women	87	57 (66.6)	0.63 (0.36-1.09)	0.10
*Visit (weeks of gestation)*				
Late >18 weeks of gestation	375	257 (68.5)	1	
Early ≤18 weeks of gestation	133	113 (85.0)	2.59 (1.53-4.38)	0.00
*Availability of SP*				
No	54	22 (40.7)	1	
Yes	454	348 (76.7)	4.78 (2.66-8.57)	0.000
Distance to health facility				
>60	15	7 (46.7)	1	
31–60	153	101 (66.0)	1.26 (0.92-1.74)	0.11
≤30 min	340	262 (77.1)	1.45 (0.16-1.61)	0.14
*Influence to make visit*				
Friends/radio	20	8 (40.0)	1	
Husband	91	69 (75.85)	1.48 (1.97-2.07)	0.002
Self	397	293 (73.8)	1.59 (1.51-4.47)	0.003
Parity				
0	170	126 (74.1)	1	
1–3	314	234 (74.5)	1.01 (0.10-1.60)	0.07
4+	24	10 (41.7)	0.244 (0.10-1.59)	0.09
*Gravity*				
Primigravidae	223	150 (67.3)	1	
Secondgravidae/multigravidae	285	220 (77.2)	1.26 (1.20-1.59)	0.05

After controlling for potential confounder, only two factors were associated with timely up take of first dose of SP; early antenatal booking [AOR = 2.59; 95 % CI 1.51–4.46; P = 0.001] and availability of SP at the facility [AOR = 4.63; 95 % CI 2.51–8.54; P < 0001] Table 4.

**Table 4:** Multivariate analysis on factors associated with timely uptake of the first dose of SP (n = 508)

Variable	AOR (95 CI %)	P value
*Visit (weeks of gestation)*		
Late >18 weeks	1	
Early ≤18 weeks	2.59 (1.51–4.46)	0.001
*Availability of SP*		
No	1	
Yes	4.63 (2.51–8.54)	<0.0001
*Gravity*		
Primigravidae	1	
Secondgravidae/multigravidae	1.16 (0.99–1.59)	0.07
*Influence to make a visit*		
Friends/radio	1	
Husband	1.42 (0.97–2.07)	0.39
Self	1.17 (0.51–4.47)	0.69

None of the factors were significantly associated with timely uptake of a second dose of SP Table 5.

**Table 5:** Bivariate analysis on factors associated with timely uptake of a second dose of SP (n = 438)

Variable	Total received second dose of SP (n = 438)	Timely uptake second dose of SP (n = 400)	OR (95 % CI)	P value
		No. (%)		
*Age (years)*				
14–17	7	7 (100)	1	
18–35	385	353 (91.7)	0.61 (0.24–1.53)	0.29
36–44	46	40 (87.0)	0.01 (0.00–1.03)	0.99
*Marital status*				
Not married	82	73 (89.0)	1	
Married	356	327 (91.9)	1.39 (0.63–3.06)	0.41
*Level of education*				
No formal	40	38 (95.0)	1	
Primary school	290	265 (91.4)	0.83 (0.39–1.75)	0.33
Secondaryschool/college	108	97 (89.8)	0.46 (0.09–2.19)	0.44
*Occupation*				
Farmer	157	150 (95.5)	1	
Petty business women	207	185 (89.4)	0.86 (0.38–1.96)	0.72
Business women	74	65 (87.8)	0.34 (0.12–1.94)	0.08
*Visit (weeks of gestation)*				
Late >18 weeks	321	290 (90.3)	1	
Early ≤18 weeks	117	110 (94.0)	1.68 (0.71–3.93)	0.24
*Availability of SP*				
No	8	6 (75.0)	1	
Yes	430	394 (95.5)	3.64 (0.71–18.73)	0.12
*Time of walking to health facility*				
>60	9	7 (77.8)	1	
31–60	133	121 (91)	1.34 (0.06–1.86)	0.15
≤30 (minutes)	296	272 (91.9)	1.38 (0.61–1.57)	0.21
*Influence to make visit*				
Friends/radio	15	14 (93.3)	1	
Husband	79	69 (87.3)	0.42 (0.97–2.07)	0.07
Self	344	317 (92.2)	1.12 (0.51–4.47)	0.09
*Parity*				
0	145	131 (90.3)	1	
1–3	271	251 (92.6)	1.08 (0.14–1.62)	0.24
4+	22	18 (81.8)	0.35 (0.11–1.61	0.08
*Gravity*				
Primigravidae	307	283 (92.2)	1	
Secondigravidae/Multigravidae	131	117 (89.3)	0.41 (0.37–2.82)	0.33

### Factors associated with timely uptake of discount voucher for ITN

Various factors were associated with timely uptake of discount voucher: early antenatal booking [OR = 206; 95 % CI 83.15–512.84; P < 0.000], multigravidae [OR = 0.45; 95 % CI 0.28–0.71; P = 0.01]. Women with a child [OR = 2.18 (1.39–3.39); P = 0.001] Table 6.

**Table 6:** Bivariate analysis on factors associated with timely uptake of discount voucher n = 486

Variable	Total received discount voucher (n = 486)	Timely uptake of discount voucher (n = 104)	OR (95 % CI)	P value
		No. (%)		
*Age (years)*				
14–17	8	3 (37.5)	1	
18–35	427	95 (22.2)	0.47 (0.19–1.12)	0.9
36–44	51	6 (11.8)	0.22 (0.04–1.17)	0.08
*Marital status*				
Not married	85	21 (24.7)	1	
Married	401	83 (20.7)	0.79 (0.46–1.37)	0.44
*Level of education*				
No formal	48	9 (18.8)	1	
Primary school	324	67 (20.7)	1.25 (0.75–2.07)	0.389
Secondary school/college	114	28 (24.6)	1.41 (0.61–3.27)	0.42
*Occupation*				
Farmer	177	33 (18.6)	1	
Petty business women	230	54 (23.5)	1.86 (0.38–1.96)	0.72
Business women	79	17 (21.5)	1.19 (0.62–2.31)	0.59
*Visit (weeks of gestation)*				
Late >18 weeks	360	6 (1.7)	1	
Early ≤18 weeks	126	98 (77.8)	206 (83.15–512.84)	<0.0001
*Gravity*				
Primigravidae	139	44 (31.7)	1	
Secondgravidae/multigravidae	347	60 (17.3)	0.45 (0.28–0.71)	0.01
*Time of walking to health facility*				
>60	16	3 (18.8)	1	
31–60	149	23 (15.4)	0.72 (0.20–2.59)	0.61
≤30 min	321	78 (24.4)	1.26 (0.33–4.79)	0.73
*Influence to make visit*				
Friends/radio	17	1 (5.9)	1	
Husband	86	15 (17.4)	1.21 (0.03–1.60)	0.13
Self	383	88 (23.0)	1.29 (0.04–2.41)	0.26
*Parity*				
0	160	49 (30.6)	1	
≥1	326	55 (18.0)	2.18 (1.39–3.39)	0.001

After controlling for potential confounders, only one factor was found to predict timely uptake of discount voucher; which was Early antenatal booking [AOR = 200; 95 % CI 80.38–4.98; P < 0.0001] Table 7.

**Table 7:** Multivariate analysis on factors associated with timely uptake of discount voucher (n = 486)

Variable	AOR (95 % CI)	P value
*Visit (weeks of gestation)*		
Late >18 weeks	1	
Early ≤18 weeks	200 (80.38–498)	<0.0001
*Gravidity*		
Primigravidae	1	
Secondgravidae/multigravidae	0.56 (0.25–1.24)	0.15
*Parity*		
0	1	
≥1	1.85 (0.19–3.75)	0.83

## Discussion

This study shows a higher prevalence of SP (IPTp) uptake, compared to that reported from a previous study [5]. The difference might be due to the current study site being along the lake zone with high prevalence of malaria,therefore peoples’ attitude and perception differ on the importance of adhering to malaria prevention intervention for pregnant women.

The majority of women 86 % in this study used two doses of SP IPTp, this is a higher proportion than that reported in another study;whereby, the uptake was 32 % [5]. The increase in percentage of women who use SP for IPTp may be a result of continued advocacy and sensitization carried out in the country by various stakeholders under NMCP. Reasons given for not using two doses are the same as those reported in another previous study [8].

Proportion of women who received first dose of SP timely and second dose differed. The observed difference might be due to the fact that majority of the study population made their first visit late and hence affected timely uptake of the first dose and not the second dose. In additions the percentage of women who used first dose timely was higher in this study as compared to that reported in the study done in North east Tanzania [7]. The difference might be due to the fact this study was done in area of high malaria transmission, so peoples’ attitude and perceptions on malaria prevention differ.

The factors which influence early uptake of the first dose of SP included early antenatal booking, availability of SP at a facility, second/multigravida and self decision to make a visit; different results were observed by other authors [8–10] where, timely uptake of the first dose was only determined by health care facility factor and no factor was based on individual characteristic. The main reason for this difference might be due to the fact that this study was done in area of high malaria transmission so perception and attitude differ.

From this study, none of the factors were associated with timely uptake of the second dose of SP similar to another study done in Malawi [9].

Slightly, more than three quarters of pregnant women received optimal protection time after using SP IPTp. Optimal protection time only occurs if a pregnant women receives two doses as the guideline suggests. Although a high proportion of pregnant women received two doses, low proportion received optimal protection time because majority of women started antenatal visit late.

Tanzania has been implementing a discount voucher system at a national level since 2004, to deliver insecticide treated nets to pregnant women. Although relatively simple, this process involve a sequence of steps that a woman has to attend to ANC, receive the discount voucher and also redeem it for an ITN [10–14]. Findings from this study shows that a higher proportion of pregnant women received discount voucher, this is higher than that observed in Ghana [11] where only 50.7 % of pregnant women received discount voucher. The observed difference might be due to the fact that in Tanzania there is a strong national commitment on distribution of ITN among pregnant women. The reasons given for those who did not receive discount voucher is the same as that observed from previous study [11]. Timely uptake of discount voucher was low, possibly due to the fact that majority of the study population made their first ANC visit late.

Majority of study participants who received discount voucher redeemed for an ITN. This is a different observation from previous studies where very few women redeemed for an ITN [11, 15–17]. The observed difference is probably due to the fact fact that, other studies were done in the rural locations where social economic status of women is relatively poor. In the minority who were unable to redeem for ITNs, the main reasons for redemption failure included; failure to know the shops where they could get ITN, lack of money to top u and one woman reported to have a bed net and did not like to use net obtained from discount voucher because of its unpleasant smell.

A small proportion of pregnant women received optimal protection after using ITN obtained through discount voucher. This may be due to the fact that majority of study participants received discount voucher when they were in mid way of pregnancy; around the mean time of 21 weeks of gestation. Another reason could be due to delayed redemption of discount vouchers in a significant proportion of those who timely received discount vouchers.

This study had one limitation, information on the uptake of SP (IPTp) was collected through a self report method; no blood drug levels were measured. However antenatal records were used to validate uptake.

In conclusion, this reveals that the timely uptake of malaria preventive interventions and receiving malaria optimal protection time depended on early antenatal booking and availability of SP and ITN voucher at the facility.

Therefore it is recommended that the Ministry of Health should ensure that these services are available at the facility all the time.
